# Beadchip technology to detect DNA methylation in mouse faithfully recapitulates whole-genome bisulfite sequencing

**DOI:** 10.2217/epi-2023-0034

**Published:** 2023-04-05

**Authors:** Elizabeth M Martin, Sara A Grimm, Zongli Xu, Jack A Taylor, Paul A Wade

**Affiliations:** 1 Epigenetics & Stem Cell Biology Laboratory, National Institute of Environmental Health Science, Research Triangle Park, NC 27713, USA; 2 Integrative Bioinformatics, Biostatistics & Computational Biology Branch, National Institute of Environmental Health Science, Research Triangle Park, NC 27713, USA; 3 Epidemiology Branch, National Institute of Environmental Health Science, Research Triangle Park, NC 27713, USA

**Keywords:** DNA methylation, mouse methylation array, whole-genome bisulfite sequencing

## Abstract

**Aim:**

To facilitate wide-scale implementation of Illumina Mouse Methylation BeadChip (MMB) technology, array-based measurement of cytosine methylation was compared with the gold-standard assessment of DNA methylation by whole-genome bisulfite sequencing (WGBS).

**Methods:**

DNA methylation across two mouse strains (C57B6 and C3H) and both sexes was assessed using the MMB and compared with previously existing deep-coverage WGBS of mice of the same strain and sex.

**Results & conclusion:**

The findings demonstrated that 93.3–99.2% of sites had similar measurements of methylation across technologies and that differentially methylated cytosines and regions identified by each technology overlap and enrich for similar biological functions, suggesting that the MMB faithfully recapitulates the findings of WGBS.

Methylation of the 5′ position of cytosine in DNA is one of the most highly studied epigenetic marks in humans. It has been profiled across disease states, environmental exposures and age [1–6]. However, identifying consistent biomarkers of exposure [7], effect [8] and disease [9] across populations has resulted in limited translation to understanding causal biological mechanisms [10,11]. The dearth of correlations between biomarkers of epigenetic change and mechanisms of epigenetic change in disease is likely to result from features inherent to human population research such as mixed genetic backgrounds, poor availability of target tissue and variable environmental conditions [12–15].

These issues may be overcome by changing the paradigm and studying DNA methylation in a highly controlled system where genetics, environment and tissue availability are controlled by the researcher [16]. Laboratory mice, as a model system, offer many of these advantages. However, assessment of methylation in mouse studies has been dependent on more costly and bioinformatically challenging technologies such as whole-genome bisulfite sequencing (WGBS) and reduced-representation bisulfite sequencing (RRBS) [17]. Recently, Illumina released the Mouse Methylation BeadChip (MMB), which assesses DNA methylation at 297,415 sites across the mouse genome. This array has been used to catalog DNA methylation patterns in multiple mouse tissues and to identify signatures associated with aging and tumorigenesis [18,19]. Additionally, Fennell *et al.* compared the MMB array to RRBS and found that data collected by the array are comparable to those collected by RRBS [20]. However, the authors note that there is a major tradeoff between technologies: the MMB provides more precise measurements of methylation measurements but sequencing provides increased genomic coverage [20].

The goal of the present study was to compare the MMB array to the gold standard of DNA methylation assessment [21], WGBS, in an effort to understand the concordance between these two methods of DNA methylation assessment. This was achieved by leveraging two previously published high-depth WGBS studies of DNA methylation across autosomes [22] and the X chromosome [23] and newly performed experiments employing the MMB array. The findings presented here are unique in that they investigate the consistency of the level of methylation assessed, the overlap of differentially methylated cytosines (DMCs) and differentially methylated regions (DMRs) called by the two technologies when using the same analytical methods as well as the overlap of differential methylation called by analytical methodologies developed for each experimental technology.

## Methods

### Animal care & housing

Animals were selected to replicate the conditions from Grimm *et al.* and Duncan *et al.* [22,23]. Specifically, these studies utilized three female mice and three male mice from both the C3H/HeJ and C57BL/6N strains that were housed at Integrated Laboratory Systems (NC, USA) beginning at 8 weeks until 20 weeks of age when livers were collected. For this study, C3H/HeJ and C57BL/6J mice were purchased from Jackson Laboratories (ME, USA). They were housed in the National Institute of Environmental Health Science (NIEHS) facilities (Durham NC, ASP 2018-008). Animals were brought into the facility at 8 weeks of age and sacrificed at 20 weeks of age. Animals were maintained in climate-controlled rooms on a 12-h light/dark cycle in polycarbonate cages with filtered air. Irradiated, heat-treated hardwood bedding and cotton fiber nestlets were supplied to the mice for the duration of the study. NIH31 and reverse osmosis-treated water were provided ad libitum to all mice for the duration of the study. Animals were multihoused up to three adults per cage. For each group (C57/B6 female, C57/B6 male, C3H female and C3H male), 6 animals were assessed (n = 24) with the C57/B6 male group losing one animal during the study resulting in a total of 23 animals being assessed. Animal housing and procedures were approved by the Institutional Animal Care and Use Committee of NIEHS and followed the recommendations of the Guide for the Care and Use of Laboratory Animals [24].

### DNA extraction & bisulfite conversion

A 2-mm cube of fresh liver tissue was homogenized immediately following sacrifice into Buffer RLT plus with beta-mercaptoethanol (Qiagen, CA, USA). DNA was then extracted with the Qiagen Allprep DNA/RNA/Protein Mini kit according to the manufacturer's protocol. Following extraction, 500 ng DNA per sample was bisulfite converted using the EZ Gold DNA methylation kit (Zymo Research, CA, USA) following the manufacturer's instructions. To assess technical replicates across arrays, an animal from each group was assessed on each of the four arrays used to measure methylation (n = 16) for a total of 35 samples assessed across all arrays. DNA was then processed through the Infinium array protocol (Illumina, CA, USA). Array BeadChips were scanned on the Illumina iScan system to produce IDAT files.

### Preprocessing & statistical analysis

Mouse array IDAT files were preprocessed using ENMix [25] with the following parameters: the out-of-band method was used to estimate background normal distribution parameters; RELIC-based dye correction was applied; quality control (QC) was performed to exclude low-quality samples; and interarray quantile normalization was performed (Supplementary Figure 1). A total of 5216 probes were identified as low-quality CpGs by poor detection p-values (p > 0.05) and were removed from subsequent analysis. A total of 280,900 probes were summarized into beta values. Data were visualized using density distributions during all preprocessing steps. The 7364 probes described in Zhou *et al.* as those that were designed to target multiple regions of the genome were not masked nor were the subset of 4541 that were described as suboptimal [18]. Instead, the performance of these probes relative to WGBS was performed and when they were removed during processing was evaluated. They are referred to as multimapping probes throughout the remainder of the text. During beta value summarization, 1221 probes identified to be multimapping were removed.

To assess the reliability of the mouse methylation array, technical replicates from each group were assayed (i.e., a C57/B6 female, a C57/B6 male, a C3H female and a C3H male) across all four arrays used. Regression and intraclass correlation (ICCs) analyses were then performed to assess the agreement of each of the four technical replicates across the arrays.

Postprocessed methylation counts from Grimm *et al.* and Duncan *et al.* [22,23] were utilized to summarize percent methylation values for this study (GSE106379, GSE106208). These studies utilized a read depth of 150× genomic coverage. Of the WGBS data collected for the 12 animals in the previously published studies, an average of 99.5% of the 19,751,474 sites had nonzero read counts per animal.

### Comparison of WGBS & array methylation

To compare the newly generated array DNA methylation data to published WGBS data [22,23], CpG sites queried by the array were first converted from mm10 to mm9 coordinates with the USCS liftover utility to match genome builds between postprocessed methylation counts and beta values. A total of 484 probes were removed during this conversion. Next, probes were filtered to retain only sites confirmed to be in CpG context sites (i.e., non-SNP sites) across both genetic backgrounds removing a total of 5127 additional probes (of which 1904 were multimapping probes) and leaving a total of 275,289 probes for analysis (Supplementary Table 1). This set of beta values was compared with average percent methylation values for all sites to determine whether a locus was differentially methylated between WGBS and the MMB. The number of probes identified as differentially methylated and the overlap to multimapping probes was identified at 10% intervals (Supplementary Table 2). This enabled an understanding of how DNA methylation is measured differently between the two technologies and allowed the determination of whether these probes had been previously identified as multimapping probes. Next, any sites containing missing data were removed prior to applying formal statistical methods leaving 273,474 sites. Comparability of WGBS data and array data was determined by principal component analysis using singular value decomposition to observe the separation for methylation values by methodology (sequencing vs array) and biological factors (sex and strain).

### Identification of DMCs & DMRs

Once major drivers of variation were assessed, analysis of the data by supervised methods was undertaken on the 273,474 high-quality nonmissing probes [18]. First, the same analytical method (limma) was applied to understand whether the two different experimental methods (sequencing vs array) would generate similar results. Limiting the analyses to only those sites assessed by both technologies allowed for the normalization of the false discovery rate correction, which would have been much higher for WGBS, as it assessed 100 times more sites than the MMB [22,23]. DMCs associated with either sex or strain but not technology were identified by limma [26] using beta values as the raw inputs for the MMB and methylation percentages as the raw inputs for WGBS. Statistical significance was originally set at q < 0.05. However, after further analyses of the data, the additional criterion of a beta difference >|0.2| was added to focus on those CpGs that were highly different [27]. The models used examined each cytosine individually to assess whether it was associated with sex or strain independently, while the other biological variable was included in the model as a covariate. To identify DMRs, metilene [28] was run with a DMR being defined as 3 or 5 CpGs located within 1 kb of each other, having a minimum change in methylation of either 5% or 20% and a q-value < 0.05. The definitions for the amount of methylation change needed was varied, as 5% is the recommended parameter by metilene, however, 20% has been used in prior assessments [22,23]. To understand what additional data is provided by sequencing data relative to array (as it assesses a larger number of sites), the DMR analysis for sequencing data was repeated using additional methylation information from 1 kb regions centered around the probe.

While these analyses allowed for direct comparison of the two methods, identification of the comparability of the array and sequencing methods when using analysis methods designed specifically for each experimental platform was also a goal. To address this question, DMCs were identified using an epigenome-wide association study (EWAS) that applied *reffreeewas* [29] as this method is among the most widely used to assess DMCs as measured by arrays. Next, DMRs were identified by metilene and DSS [30] using the WGBS data as both methods are regularly used to assess differential methylation for sequence data. Lastly, to evaluate comparability between sites and regions, the number of the DMCs called from the array data found in the DMRs called from the sequencing data were assessed and gene ontology analysis was performed using GREAT to understand the biological functions of these sites [31].

## Results

To address how well the Illumina array compares to the current gold-standard measure of DNA methylation, WGBS, liver tissues were collected from five C57BL/6J male mice, six C57BL/6J female mice (in the remainder of the text, these animals are referred to as B6 males/females), six C3H/HeJ male mice and six C3H/HeJ female mice (in the remainder of the text, these animals are referred to as C3H males/females). Animals were aged in a controlled environment from 8 to 20 weeks of age, sacrificed at identical ages and liver samples collected to mirror the previously generated DNA methylation dataset. Bisulfite conversion was performed and the newly developed Illumina Mouse Methylation array was utilized to assess DNA methylation.

### Methylation variability at CpG level

**Figure 1.**
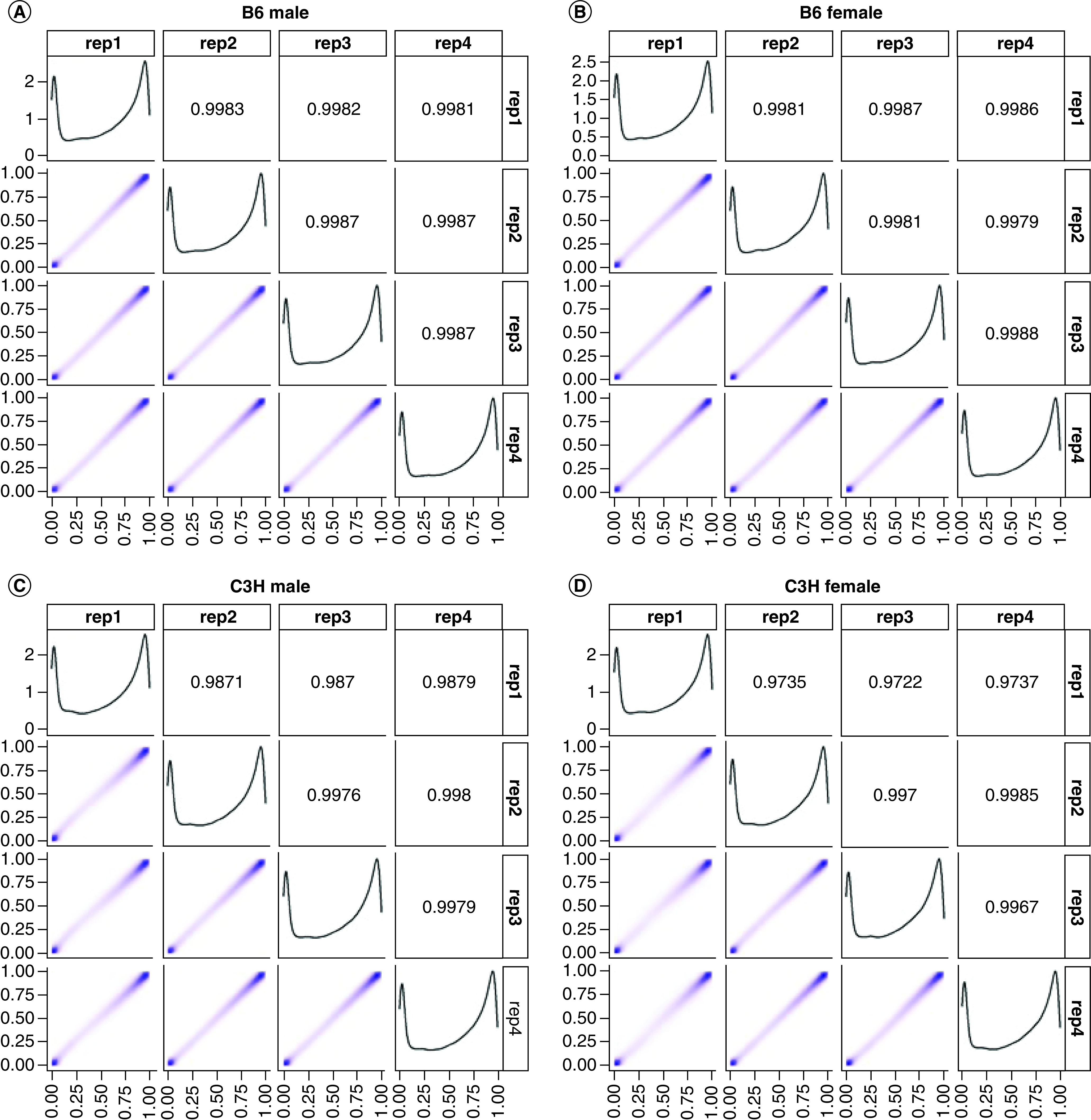
Correlations between technical replicates for all four technical replicates for each group. **(A)** B6 males, **(B)** B6 females, **(C)** C3H males and **(D)** C3H females.

Interarray variability was assessed in the data as prior work has shown that position and array can affect methylation readouts. One animal from each group was included across each of the four slides used during the experiment. The readouts between arrays were highly consistent with samples replicating well across arrays (Figure 1). However, it seems that the B6 readings (Figure 1A & B) were slightly more consistent than the C3H readings (Figure 1C & D). Additionally, as with human methylation, a bimodal distribution of methylation was identified: most probes being either at the high or low end of beta values with intermediate levels of methylation being far less common. Additionally, ICCs were calculated for each probe to determine the replicability in a locus-specific manner (Supplementary Figure 2 & Supplementary Table 1). The distribution of ICCs was similar to distributions of beta values derived from human populations [32,33].

### Comparison of WGBS to Illumina array

**Figure 2.**
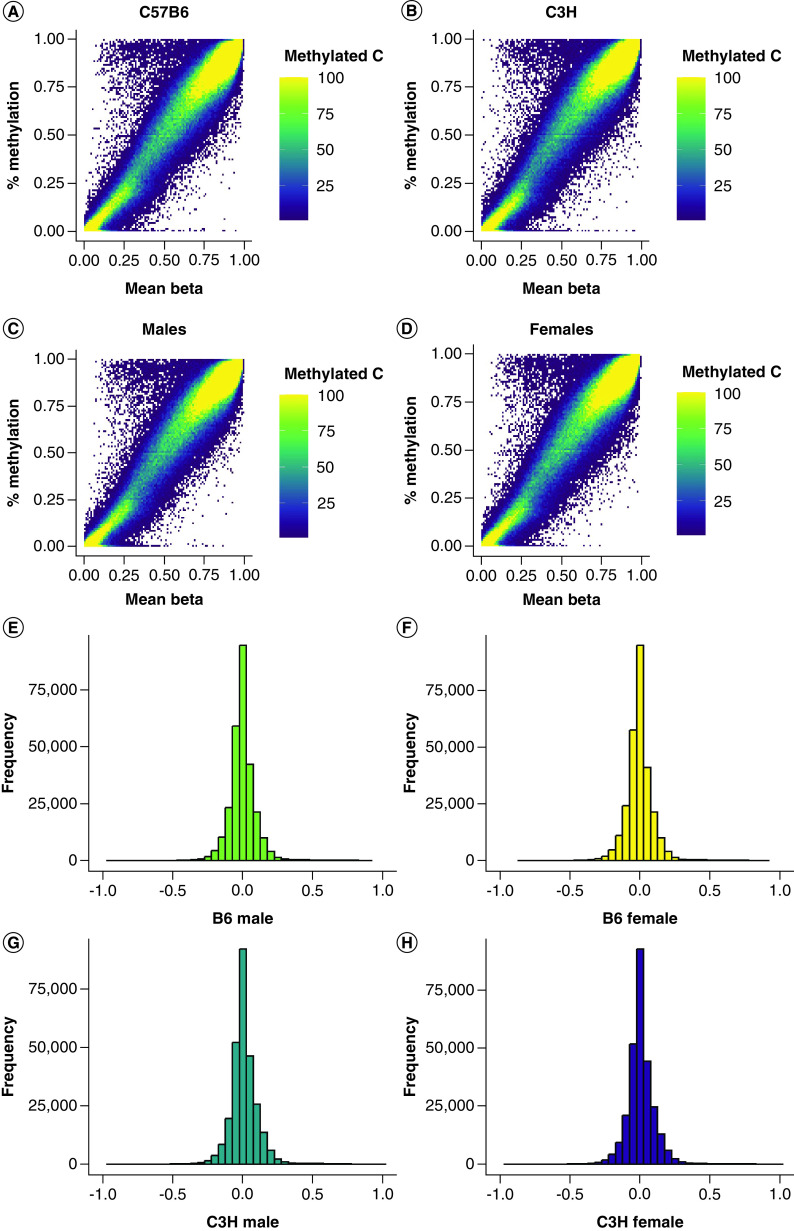
Correlations of weighted methylation values generated by WGBS data and beta values generated from array image data. All sites with each genetic background **(A)** C57B6 (B6) mice **(B)** C3H/H3J (C3H) as well as each sex **(C)** males and **(D)** females were considered during correlation analysis. Beta values calculated from array image data are plotted along the x-axis while % methylation calculated from WGBS data is plotted along the y-axis. In addition, the number of sites are plotted by difference in % methylation values and beta values for each group assayed in the experiment **(E)** B6 males, **(F)** B6 females, **(G)** C3H males and **(H)** C3H females.

After QC and normalization of the array data (see Methods section), both the WGBS and map coordinates supplied by Illumina's manifest file were placed on mm9 for direct comparison. The mean methylation level within each group was then calculated for WGBS and the average beta value within each group (Figure 2A–D). Generally, 6.7% of sites were outside a 10% difference in methylation across all conditions (Figure 2E–H & Supplementary Table 2). At a 20% difference in methylation between the two technologies, only 1.2% of sites are called as different and at 30%, only 0.8% of sites are assessed as different by the two technologies. Interestingly, the probes found to be different between WGBS and the MMB were not predominated by probes identified by Zhou *et al.* to be masked. At a 10% difference in methylation, only 11.4% of sites called as different between the two technologies represent multimapping probes. This percentage increased as the difference in methylation increased: at 20%, 45.5% of sites are multimapping probes and at 30% of difference, 70.3% of sites are multimapping probes. This data suggests that the probes identified as different between WGBS and the MMB are not simply capturing multimapping probes but are identifying regions of the genome that are assayed differently by the two technologies.

**Figure 3.**
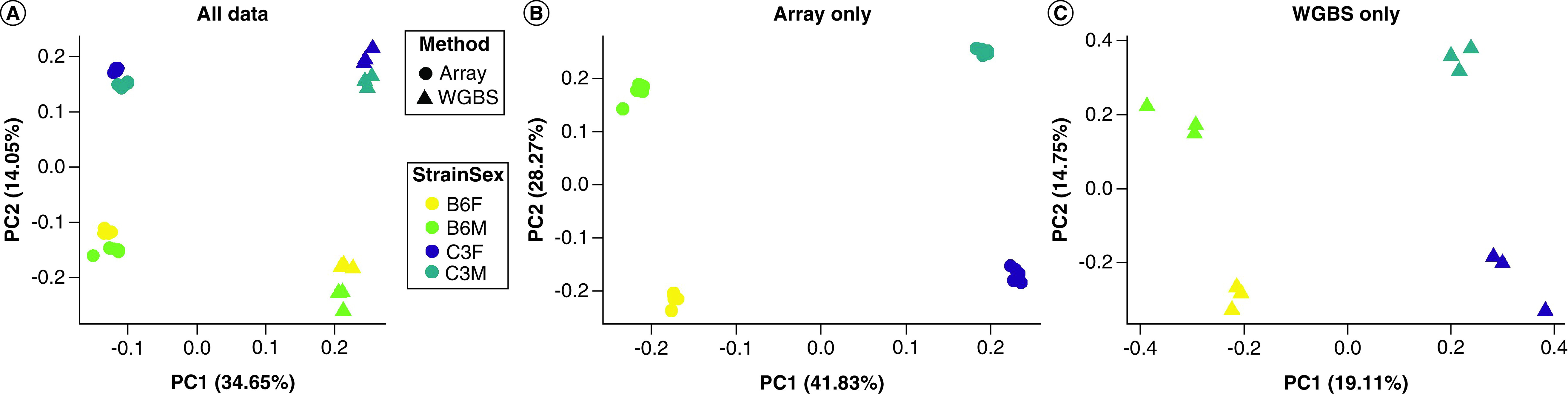
Principle component (PC) plots for identifying key factors along which data separated. **(A)** All data are shown together and demonstrate separation primarily by method (PC1) and then by strain (PC2). When considering the experimental methods separately, the array **(B)** and WGBS **(C)** showed separation primarily by strain (PC1) and secondarily by sex (PC2).

Next, major sources of variance across all data were determined. When comparing methylation values obtained by the array to those obtained by sequencing, the first principal component of separation accounting for 37% of the total variance in values was due to the experimental method, with strain driving an additional 15% of the variance (Figure 3A). Importantly, the experimental methods were assessed at different time points and in different animals so some variation may be due to the batch effect. Once data were separated by the experimental method, a similar separation was observed along the first principal component by strain with the second component accounting for separation by sex (Figure 3B & C). This separation was clearer for the array than it was for the sequencing data. To address this formally, ICCs were calculated for array data and sequencing data. The average of the probe ICCs for the array was 0.361, which was higher than for the sequencing data, which had an average probe ICC of 0.198, suggesting an increase in the precision of measurement by the array compared with sequencing.

Given the concordance of the experimental methods in separating groups by principal components, the performance of the MMB and its ability to identify differential methylation compared with WGBS was also of interest. Therefore, DMCs and DMRs were assessed between sex and genetic background using the same method (limma and metilene, respectively) and methods developed for each data type (reffreeewas metilene and DSS) and the overlap between these two analytical methods was compared between different measurement techniques.

### Assessment of data by the same method

**Figure 4.**
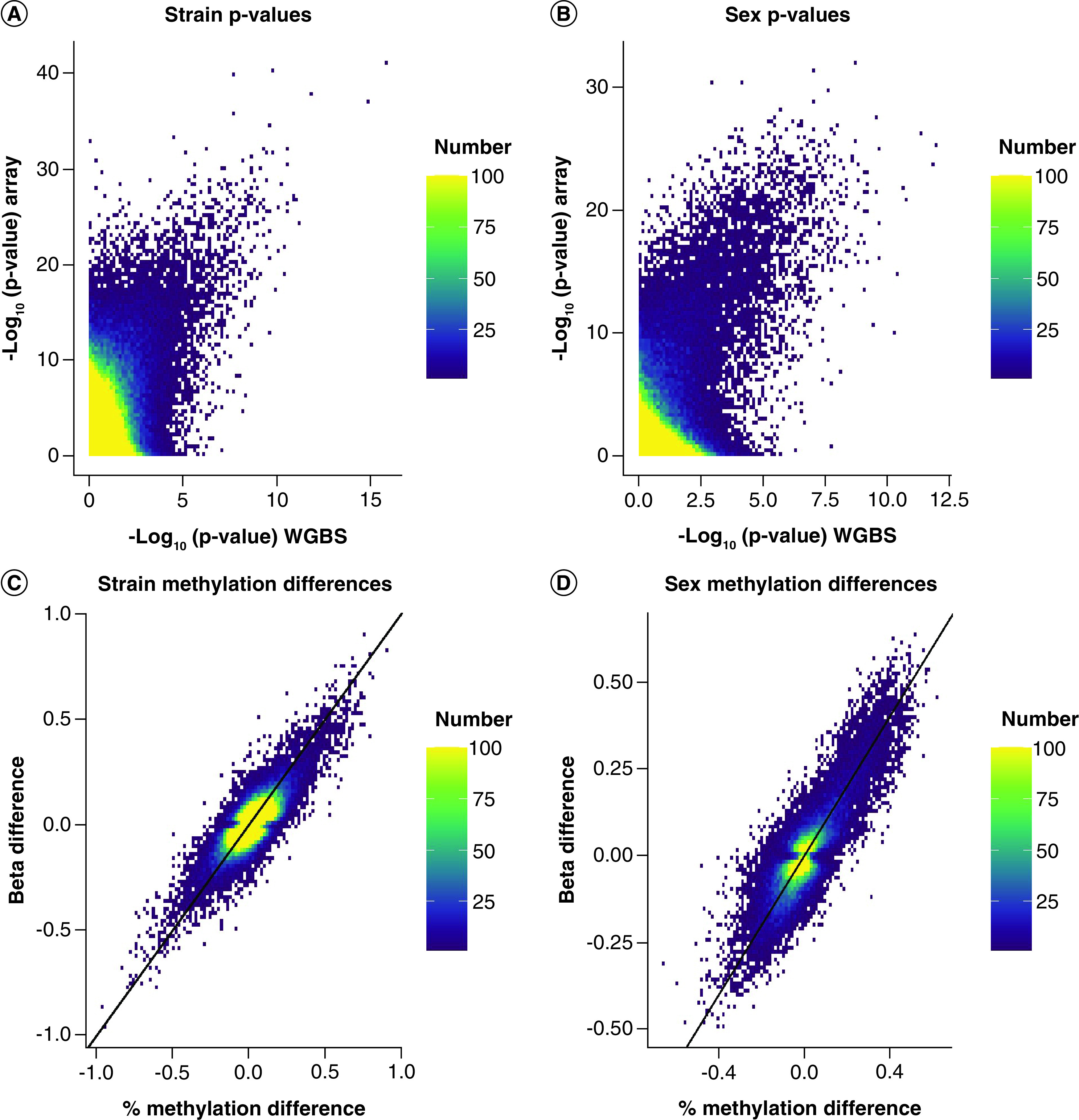
Scatter plot of p-values generated by limma and differences in methylation detected by the two technology. The distribution of p-values by experimental methodology generated by the same statistical model limma were compared for **(A)** strain and **(B)** sex. As a secondary comparison, scatter plots of methylation differences were generated for all significant sites (q < 0.05) for **(C)** strain and **(D)** sex to compare experimental methodologies.

To identify DMCs in WGBS and MMB data, limma [26] was utilized for all high-quality nonmissing sites (n = 273,474); p-values were calculated interpedently for the strain comparison (Figure 4A) and sex comparison (Figure 4B). The correlation between p-values improved as the level of significance increased. Although the overall correlation between p-values was low (R^2^ = 0.01022 for sex and R^2^ = 0.02943 for strain), the correlation between p-values improved as the level of significance increased. Interestingly, notable differences in the magnitude of p-values were evident regardless of the variable being considered.

The concordance of methylation for any site called as statistically significant by the traditional metric of q <0.05 (Figure 4C & D) was then assessed. Surprisingly, a much higher degree of concordance of the difference in methylation called at these sites was observed as indicated by the tightness of correlation along the x = y line (R^2^ = 0.6236 for strain and R^2^ = 0.7388 for sex). These data overall suggest that the two experimental methods detect similar differences in methylation at cytosines but the data variability results in differences in the calculated p-values. This increase in p-values is likely related to an increase in consistency in methylation measurement by the array as supported by the higher average ICC for the array relative to sequencing.

**Figure 5.**
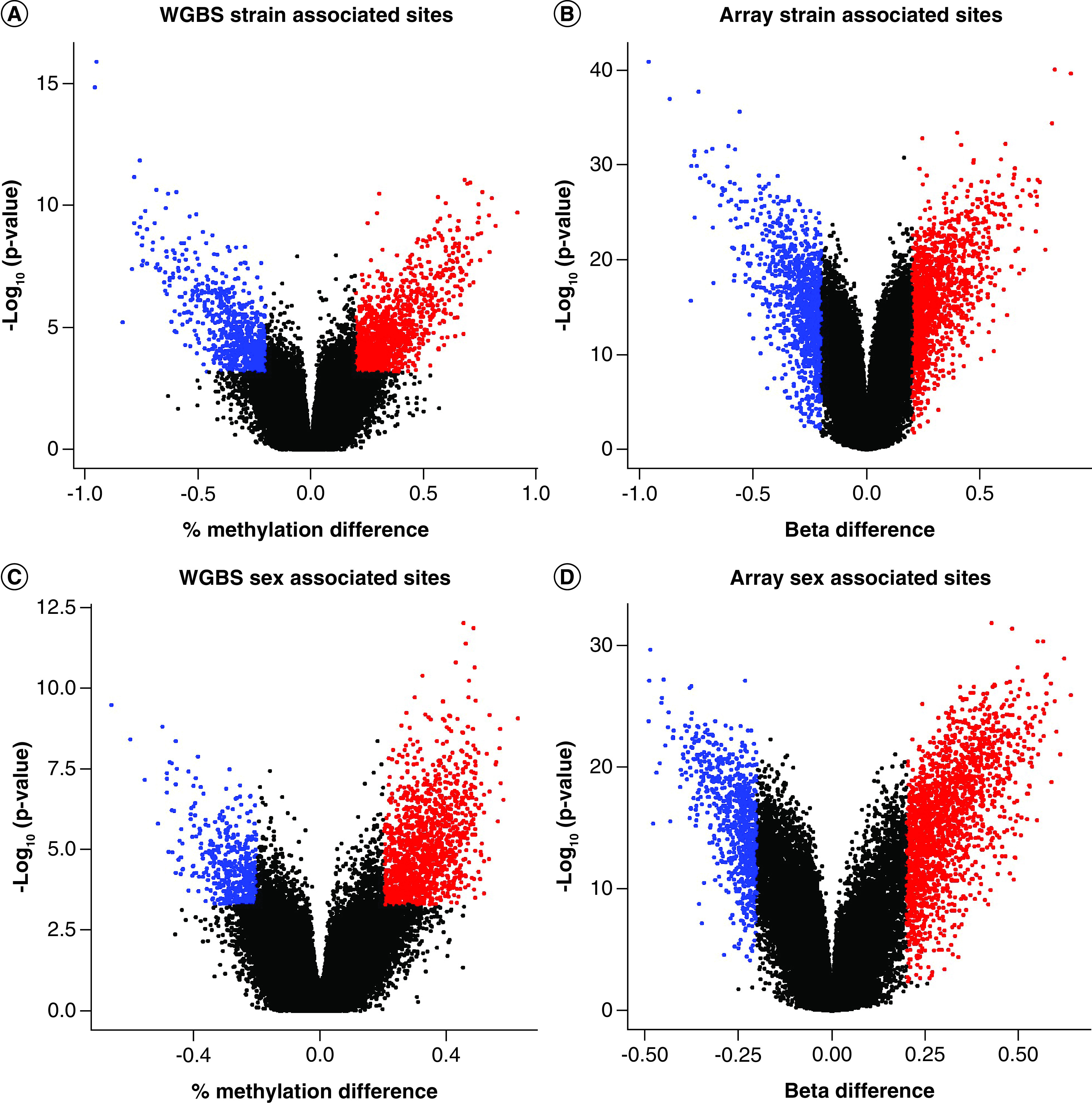
Volcano plots noting sites determined to be differentially methylated by limma for strain by (A) Whole Genome Bisulfite Sequencing (WGBS) and (B) the mouse methylation beadchip (MMB) array as well as for sex by (C) WGBS and (D) array. The -log10(p-value) is displayed on the x-axis while the difference in percent methylation and beta value for each group is denoted along the y-axis. Blue dots indicate DMCs that were higher in the referent group by at least 20% while red dots indicate DMCs that were higher in the referent group by at least 20%.

To further limit the analysis to high-confidence sites [27] that are more likely to influence biological differences, a more stringent statistical threshold for significance of q < 0.05 and an average methylation difference >20% was imposed across the 273,474 sites analyzed. For the sequencing data, a total of 2344 DMCs (∼0.8% of all sites analyzed) were identified with 861 (36.7%) displaying lower methylation in B6 relative to C3H and 1483 (63.3%) displaying higher methylation in B6 relative to C3H (Figure 5A & Table 1). From the array data, a total of 2983 DMC sites (~1% of all sites analyzed) were associated with strain-based differences (Figure 5B & Table 1). Of the 2983, 1694 (56.8%) displayed higher methylation levels in B6 relative to C3H mice while 1289 (43.2%) displayed lower levels in B6 relative to C3H (Table 1). Across both methods, 531 sites (32.7% of all 1619 sites identified as less methylated in B6 relative to C3H by either method) were called by both methods and 847 sites (36.3% of all 2330 sites identified as more highly methylated in B6 relative to C3H) were called by both methods. Generally, roughly half of all differentially methylated sites were shared across methods.

**Table 1.** Number of differentially methylated cytosines identified through limma by each experimental methodology (array vs sequencing).

Strain					
		Sequencing			
		B6<C3H	NS	B6>C3H	Total
Array	B6<C3H	531	758	0	1289
	NS	330	269525	636	270491
	B6>C3H	0	847	847	1694
	Total	861	271130	1483	

**Sex**					
		**Sequencing**			
		**F<M**	**NS**	**F>M**	**Total**
Array	F<M	280	592	0	872
	NS	265	270088	210	270563
	F>M	0	831	1208	2039
	Total	545	271511	1418	

Results for comparisons of sex and strain across all canonical chromosomes. The number of sites are presented for higher than the referent group, not significant and lower than the referent groups by each experimental method. DMCs are designated as being significant in the comparison of strain for either C57B6 (B6) or C3H/HeJ (C3H), male (M) or female (F), and not significant in any comparison (NS).

For sex comparisons, 2911 DMCs (∼1% of all analyzed sites) were called by array while 1963 DMCs (∼0.7% of all analyzed sites) were called by sequencing (Figure 5C & D). Across both methods, there were significantly more sites called as more highly methylated in females than in males: 2039 (70.0%) versus 872 (30.0%) by array and 1418 (72.3%) versus 545 (27.7%) by sequencing. Across both methods, 280 sites (24.5% of the 1065 sites called as lower in females relative to males across either technologies) were consistently decreased in methylation in females relative to males while 1208 sites were more highly methylated in females relative to males (53.7% of the 2240 sites called as higher in females relative to males across either technology; Table 1). Similar to strain, an overlap of greater than 50% of sites were called across both technologies.

Next, metilene was used to call DMRs from WGBS data and array data independently. Parameters set for identification of DMRs were as follows: q-value < 0.05, a maximum distance of 1 kb between consecutive sites, a minimum CpG count of 3 or 5 sites and a minimum average change in methylation of 20% or 5%. Varying the minimum number of sites and the average change in methylation during DMR calls allowed observation of whether the concordance of the array and sequencing is dominated by regions where methylation is highly conserved or whether the concordance of the technologies applies to regions of the genome that are more variably methylated. When considering the most stringent of conditions that represent highly conserved regions of the genome (5 CpGs and 20% change in methylation), 19 DMRs were called for the array and 8 were called by WGBS in the comparison of the two strains (Table 2). Of these, 7 DMRs overlapped between the methodologies. In the least stringent condition, representing the more variably methylated regions of the genome (3 CpGs and 5% change in methylation), 259 DMRs were called for the array and 21 called for WGBS in the strain comparison. Of these, 19 overlapped between platforms. No overlapping DMRs were identified to have methylation levels in opposite directions. In the comparison of sex, when DMRs were defined under the most stringent conditions, there were 133 DMRs called for the array and 91 for WGBS. Of these, 90 were overlapping in the same direction. In the least stringent of comparisons, 424 DMRs were called by the array and 169 were called by WGBS. Of these, 167 were shared in both conditions. No DMRs were overlapping with opposite directions of methylation.

**Table 2.** Number of differentially methylated regions called by metilene analysis on whole-genome bisulfate sequencing and array data with the number of overlapping observations noted.

	5+ CpGs, 20%+ ΔMeth	5+ CpGs, 5%+ ΔMeth	3+ CpGs, 20%+ ΔMeth	3+ CpGs, 5%+ ΔMeth
Strain				
WGBS	8	8	21	21
Array	19	74	74	259
Overlap DMR: same direction	7	7	18	19
Overlap DMR: opposite direction	0	0	0	0
No overlap	1	1	3	2
Sex				
WGBS	91	112	136	169
Array	133	172	299	424
Overlap DMR: same direction	90	112	130	167
Overlap DMR: opposite direction	0	0	0	0
No overlap DMR	1	0	6	2

The different stringencies used for each of the methods is denoted in the columns while the total number of sites is denoted along with whether or not overlap was observed between the two experimental methods. The number and direction of overlapping DMRs called by each technology is presented.

DMR: Differentially methylated region; WGBS: Whole-genome bisulfate sequencing.

Overall, for all analyses, counts of called DMRs were generally higher for the array than the sequencing data. This is likely a function of the increased precision by the MMB over WGBS that also drove increased significance observed in the principal component analysis and DMC analysis. Interestingly, if the DMR was called from the sequencing data, there was a more than 85% chance that it was called as a DMR by the array in the strain comparison and a more than 95% chance that it was called as a DMR by the array for the sex comparison. Notably, when the sequencing data is more fully utilized by also considering all CpG sites within a 1 kb flanking region around the array CpG sites, substantially more DMRs are called. Strikingly, while there was a decrease in overlap of DMRs called by either comparison, there was still an average likelihood of 85% that a DMR called by the larger region of WGBS overlapped the DMR call from the array (Supplementary Table 3). This data demonstrates that while there is increased precision in the calls made by the MMB, it assesses a smaller proportion of the genome and may miss important changes in DNA methylation that are not represented by probes on the array.

### Assessment of data by standard methodologies

Next, an assessment of whether the same genomic loci would be identified as differentially methylated across technologies when the data was analyzed with statistical methods that were developed specifically for the data types generated by each of the experimental paradigms of methylation measurements was performed. DMCs called by reffreeewas from the array data were compared with DMRs called by metilene and DSS from the sequencing data. A total of 2572 DMCs were called as differentially methylated in the strain comparison and 2706 DMCs were called in association with sex (Table 3). As before, a roughly equal proportion of sites showed increased (n = 1103, 42.9%) and decreased (n = 1469, 57.1%) methylation in B6 relative to C3H mice in the strain comparison, while there was a skew in the sex comparison toward sites with higher methylation in females than males (n = 1860, 69.8%) relative to sites with lower methylation in females than males (n = 846, 31.2%; Table 3).

**Table 3.** Overlap of differentially methylated cytosines called from array data to whole-genome bisulfite sequencing data.

	Count (DMCs)	DSS						Metilene					
		Overlap DMR: same direction		Overlap DMR: opposite direction		Does not overlap		Overlap DMR: same direction		Overlap DMR: opposite direction		Does not overlap	
M>F	846	70	8.3%	3	0.35%	773	91.4%	54	6.4%	0	0%	792	93.6%
F>M	1860	1245	66.9%	0	0.00%	615	33.1%	1148	61.7%	0	0%	712	38.3%
B6>C3	1103	335	30.4%	0	0.00%	768	69.6%	204	18.5%	0	0%	899	81.5%
C3>B6	1469	519	35.3%	1	0.07%	949	64.6%	335	22.8%	0	0%	1134	77.2%

The number of DMCs contained within DMRs as well as percentage of overlap is noted. Comparisons that are made are male (M) and female (F), or C57B6 (B6) and C3H/HeJ (C3H).

To determine whether the called DMCs from the array were located within DMRs called from WGBS, overlap with previously published WGBS DMRs called by DSS and metilene on autosomes and the X chromosome were assessed [22,23]. Overall, DSS and metilene had similar degrees of overlap with called DMCs. The lowest overlap was seen for sites at which males had higher levels of methylation than females: 70 DMCs (8.3% of 846 DMCs called) overlapped DMRs called by DSS and 54 DMCs (6.4% of 846) overlapped DMRs called by metilene (Table 3). The highest degree of overlap observed was for sites at which females had higher levels of methylations than males: 1245 DMCs (66.9% of 1860) were contained within DMRs called by DSS and 1148 DMCs (61.7% of 1860) were contained within DMRs called by metilene. For the strain comparisons, an overlap of between ∼20–30% was observed regardless of directionality of comparison and method by which DMRs were called. This data suggests that DMRs called by DSS were slightly more likely to contain DMCs called by the array (30.4–35.3%) relative to metilene (18.5–22.8%). Across both methods, very few discordant calls between DMCs and DMRs (0–3 DMCs) were observed, suggesting that the exact overlap of calls may be driven by the tool used as both the array and sequencing generally identify similar methylation trends across the genome.

### Biology of DMCs is consistent between array & WGBS

To assess the biological function of the observed changes in DNA methylation, gene ontology analysis was performed with GREAT [31] for DMCs identified as differentially methylated for the array, separated by direction of methylation in the strain or sex comparison. DMCs identified as having higher levels of methylation in males relative to females are affiliated with differences in histone modification (Supplementary Table 4). Specifically, they are associated with regulation of histone H3K36 methylation, histone H3K4 methylation and histone H2A ubiquitination. Similar pathways are observed as enriched at DMCs where methylation levels are also higher in females versus males. In contrast to the observed consistency in the comparison of males versus females, strain-specific differences are not as similar between those that are more highly and lowly methylated. Interestingly, strain differences appear to enrich for processes related to metabolism and body shape. Mitochondrial transport, detection of stimulus and glycolytic processes were associated with DMCs that are higher in C3H relative to B6. However, DMCs with higher methylation in B6 relative to C3H were affiliated with carboxylic acid metabolic process, fatty acid/lipid metabolism and response to insulin. Overall, these results indicate that differences between males and females are highly consistent while DMCs identified as strain-dependent show differential biological enrichment that is dependent on the direction of methylation.

## Discussion

Overall, the findings presented here show the MMB array is a robust platform that faithfully recapitulates major aspects of methylation patterns seen with the current gold standard, WGBS. The array technology allows for the potential to study DNA methylation in a mechanistic way that is more easily analyzable than sequencing. For this reason, we set out to validate this new technology by comparing newly generated MMB array data to previously collected WGBS sequencing data. This is supported by the consistency between the methylation levels captured by array and those captured by WGBS and the consistency of methylation changes and biological function observed by both technologies in association with the biological variables of sex and strain. Strikingly, the work presented here details an increase in statistical power by the MMB relative to WGBS when assessing methylation differences. Together, these findings suggest that the MMB is a powerful tool for assessing DNA methylation and for the assayed sites provides comparable information to that provided by WGBS.

This study demonstrates that the beta values from arrays are highly consistent with cumulative methylation values generated from WGBS data. These data support the findings from Fennell *et al.* [20] that demonstrated that RRBS and the MMB array produce comparable results. Overall, the data presented in this study suggest that the methylation values obtained by the MBB and WGBS are highly consistent across the majority (93–99%) of the assayed locations. The consistency between array and WGBS has been further supported by human literature including an earlier paper that demonstrated that normalization of DNA methylation data of array beta values faithfully recapitulates average methylation values obtained from WGBS in humans [34,35]. Furthermore, recent studies have demonstrated that the Illumina EPIC arrays and methyl capture sequencing in humans also produce similar results [36,37]. These results suggest that the MMB faithfully recapitulate overall genomic patterns observed when utilizing WGBS.

In addition to the recapitulation of directionality of methylation change, the analysis presented here revealed that the MMB has the power to clearly separate animals by biological variable. This replicates the separation by biological variable observed in the WGBS data [22,23]. Comparing this data with other studies that have utilized arrays, differences by sex of this magnitude have been captured in human populations [38–42]. However, the differences observed by strain bring to light a unique feature of studying inbred mouse lines: they can be used to study genetic contributions to epigenetic differences. This is a unique opportunity as methylation level at SNPs has been a consistent source of variation in human studies that has been difficult to address [43–46]. Beyond identifying key biological differences, the results also suggest that the number of sites identified as different is higher in inbred mouse populations than the number observed in human population studies [47]. This large effect size is likely the result of the controlled nature of both the environment and genetic backgrounds that is only present in animal studies and not present in human populations. These results highlight unique opportunities to study the impact of fundamental biological variables such as sex and genetic background on epigenetic profiles in the context of exposure, aging and disease.

Interestingly, the MMB has an increased number of statistically significant probes associated with biological variables relative to WGBS. In addition to showing separation by biological variables by both WGBS and MMB data, the data presented here also demonstrate a concordance between calls made by WGBS and MMB at both DMC and DMR levels. However, statistical significance differs between the calls made by the same method for each technology despite agreement in beta differences and differences in cumulative methylation. This may be related to the observed increase in precision from MMB relative to WGBS as demonstrated by ICC, a finding also presented in Fennell *et al.* [20]. One hypothesis for the increase in precision is the potential ability of the array to sample more alleles than WGBS: 500 ng of genomic DNA theoretically has 2.85 × 10^19^ moles of genome, or 10^5^ copies of each allele going into the array amplification reaction compared with 10 s of copies of each allele going into the WBGS. Regardless of source, the increase in consistency of the MMB over WGBS appears to be a driving factor in yielding more significant DMC and DMR calls. Despite this increase in precision, WGBS has its own strengths relative to the array. While the array nicely recapitulates methylation patterns at the regions that it assays, sequencing can assess much more of the genome than the array. This is demonstrated by the finding that significantly more DMRs were called when a 1 kb flanking region was included in the DMR calls. In addition, Grimm *et al.* and Duncan *et al.* were able to assess 19,751,474 methylated cytosines, while the array captured 273,474 methylated cytosines. The amount of information captured by WGBS comes at the cost of decreased precision. Taken together, these results suggest that for sites assessed by the array, differences in methylation are more easily detected.

While this study demonstrates that WGBS and the MMB recapitulate genomic patterns, it is not without its limitations. While we utilized the same sex and strains in the analysis of DNA methylation, there have been many reports of differing results arising from different lab environments [48,49]. Given that the tissues in this study were not derived from the same mice at the same time, some results we attributed to differences between the array and WGBS could be attributed to differences in experimental animals. However, this may suggest that the findings presented here underestimate the concordance between the MMB and WGBS [50]. In addition to different animals, we focused on highly changed loci by utilizing a threshold of a beta difference >0.2, which is based on human studies [27]. Empirically determining cutoff values for methylation differences for inbred genetics and controlled lab environments should be undertaken in the future. Additionally, the data presented here suggest that mice display significantly less variability in methylation patterning than human populations as indicated by the increased number of significant calls across the genome relative to those observed in human population studies that utilize array technologies [47,51,52]. For this reason, future work should focus on pipeline development for DMC and DMR calling, as using a beta difference threshold will miss small but potentially impactful changes to DNA methylation and suggest the need for different analytical methods for this context.

## Conclusion

In summary, this work details the how the Illumina MMB array replicates methylation level and significant differences in the biological comparison of sex and strain compared with WGBS. Furthermore, it demonstrates the increased precision of the MMB at the sites that it assesses, leading to more statistically significant results, and highlights the ability of WGBS to provide more information about the whole genome relative to the array. Taken together, the results presented here highlight the potential for future research to utilizes this technology and provide new understanding into the mechanisms by which DNA methylation govern epigenetic adaptation.

**Summary points**

- Assessment of DNA methylation in mice by array and whole-genome bisulfite sequencing (WGBS) produce comparable results.
- The array faithfully assesses changes in methylation across technical replicates.
- The array and WGBS can identify similarly changed differentially methylated cytosines and differentially methylated regions.
- Both technologies produce similar results in terms of locations in the genomes that are differentially methylated, though the exact degree and locations differ depending on the technology deployed.
- WGBS has more information about the entire genome but less depth in select loci present on the array.
- Biological differences detected by both technologies lay along similar pathways, though the actual locations in the genomes called as differentially methylated differ in some cases.
- Statistics for array may be beneficial to identifying differentially methylated cytosines.
- Deployment of this array will enable greater capacity to translate between human and mouse studies.

## References

[1] Jin Z, Liu Y. DNA methylation in human diseases. Genes Dis. 5(1), 1–8 (2018). 30258928 10.1016/j.gendis.2018.01.002 PMC6147084

[2] Salameh Y, Bejaoui Y, El Hajj N. DNA methylation biomarkers in aging and age-related diseases. Front. Genet. 11, 171 (2020). 32211026 10.3389/fgene.2020.00171 PMC7076122

[3] Papanicolau-Sengos A, Aldape K. DNA methylation profiling: an emerging paradigm for cancer diagnosis. Annu. Rev. Pathol. 17, 295–321 (2022). 34736341 10.1146/annurev-pathol-042220-022304

[4] Yousefi PD, Suderman M, Langdon R, Whitehurst O, Davey Smith G, Relton CL. DNA methylation-based predictors of health: applications and statistical considerations. Nat. Rev. Genet. 23(6), 369–383 (2022). 35304597 10.1038/s41576-022-00465-w

[5] Belsky DW, Caspi A, Corcoran DL DunedinPACE, a DNA methylation biomarker of the pace of aging. Elife 11, e73420 (2022). 35029144 10.7554/eLife.73420 PMC8853656

[6] Martin EM, Fry RC. Environmental influences on the epigenome: exposure-associated DNA methylation in human populations. Annu. Rev. Public Health 39, 309–333 (2018). 29328878 10.1146/annurev-publhealth-040617-014629

[7] Nwanaji-Enwerem JC, Colicino E. DNA methylation-based biomarkers of environmental exposures for human population studies. Curr. Environ. Health Rep. 7(2), 121–128 (2020). 32062850 10.1007/s40572-020-00269-2 PMC12910356

[8] Lu AT, Quach A, Wilson JG DNA methylation GrimAge strongly predicts lifespan and healthspan. Aging (Albany) 11(2), 303–327 (2019). 10.18632/aging.101684 PMC6366976 30669119

[9] Kim H, Wang X, Jin P. Developing DNA methylation-based diagnostic biomarkers. J. Genet. Genomics 45(2), 87–97 (2018). 29496486 10.1016/j.jgg.2018.02.003 PMC5857251

[10] Taryma-Lesniak O, Sokolowska KE, Wojdacz TK. Current status of development of methylation biomarkers for *in vitro* diagnostic IVD applications. Clin. Epigenetics 12(1), 100 (2020). 32631437 10.1186/s13148-020-00886-6 PMC7336678

[11] Levenson VV. DNA methylation as a universal biomarker. Expert Rev. Mol. Diagn. 10(4), 481–488 (2010). 20465502 10.1586/erm.10.17 PMC2933138

[12] Mikeska T, Craig JM. DNA methylation biomarkers: cancer and beyond. Genes (Basel) 5(3), 821–864 (2014). 25229548 10.3390/genes5030821 PMC4198933

[13] Dor Y, Cedar H. Principles of DNA methylation and their implications for biology and medicine. Lancet 392(10149), 777–786 (2018). 30100054 10.1016/S0140-6736(18)31268-6

[14] Jiang R, Jones MJ, Chen E Discordance of DNA methylation variance between two accessible human tissues. Sci. Rep. 5, 8257 (2015). 25660083 10.1038/srep08257 PMC4321176

[15] Lam LL, Emberly E, Fraser HB Factors underlying variable DNA methylation in a human community cohort. Proc. Natl Acad. Sci. USA 109(Suppl. 2), 17253–17260 (2012). 23045638 10.1073/pnas.1121249109 PMC3477380

[16] Blewitt M, Whitelaw E. The use of mouse models to study epigenetics. Cold Spring Harb. Perspect. Biol. 5(11), a017939 (2013). 24186070 10.1101/cshperspect.a017939 PMC3809579

[17] Suzuki M, Liao W, Wos F Whole-genome bisulfite sequencing with improved accuracy and cost. Genome Res. 28(9), 1364–1371 (2018). 30093547 10.1101/gr.232587.117 PMC6120621

[18] Zhou W, Hinoue T, Barnes B DNA methylation dynamics and dysregulation delineated by high-throughput profiling in the mouse. Cell Genom. 2(7), 100153 (2022). 35873672 10.1016/j.xgen.2022.100144 PMC9306256

[19] Garcia-Prieto CA, Alvarez-Errico D, Musulen E Validation of a DNA methylation microarray for 285,000 CpG sites in the mouse genome. Epigenetics 17(12), 1677–1685 (2022). 35297293 10.1080/15592294.2022.2053816 PMC9621044

[20] Fennell LJ, Hartel G, Mckeone DM Comparative analysis of Illumina Mouse Methylation BeadChip and reduced-representation bisulfite sequencing for routine DNA methylation analysis. Cell Rep. Methods 2(11), 100323 (2022). 36452869 10.1016/j.crmeth.2022.100323 PMC9701610

[21] Duge De Bernonville T, Daviaud C, Chaparro C, Tost J, Maury S. From methylome to integrative analysis of tissue specificity. Methods Mol. Biol. 2505, 223–240 (2022). 35732948 10.1007/978-1-0716-2349-7_16

[22] Grimm SA, Shimbo T, Takaku M DNA methylation in mice is influenced by genetics as well as sex and life experience. Nat. Commun. 10(1), 305 (2019). 30659182 10.1038/s41467-018-08067-z PMC6338756

[23] Duncan CG, Grimm SA, Morgan DL Dosage compensation and DNA methylation landscape of the X chromosome in mouse liver. Sci. Rep. 8(1), 10138 (2018). 29973619 10.1038/s41598-018-28356-3 PMC6031675

[24] National Research Council (US) Committee for the Update of the Guide for the Care and Use of Laboratory Animals. Guide for the Care and Use of Laboratory Animals, 8th Edition. Washington, DC, USA (2011).

[25] Xu Z, Niu L, Li L, Taylor JA. ENmix: a novel background correction method for Illumina HumanMethylation450 BeadChip. Nucleic Acids Res. 44(3), e20 (2016). 26384415 10.1093/nar/gkv907 PMC4756845

[26] Ritchie ME, Phipson B, Wu D Limma powers differential expression analyses for RNA-sequencing and microarray studies. Nucleic Acids Res. 43(7), e47 (2015). 25605792 10.1093/nar/gkv007 PMC4402510

[27] Bibikova M, Barnes B, Tsan C High density DNA methylation array with single CpG site resolution. Genomics 98(4), 288–295 (2011). 21839163 10.1016/j.ygeno.2011.07.007

[28] Juhling F, Kretzmer H, Bernhart SH, Otto C, Stadler PF, Hoffmann S. Metilene: fast and sensitive calling of differentially methylated regions from bisulfite sequencing data. Genome Res. 26(2), 256–262 (2016). 26631489 10.1101/gr.196394.115 PMC4728377

[29] Houseman EA, Molitor J, Marsit CJ. Reference-free cell mixture adjustments in analysis of DNA methylation data. Bioinformatics 30(10), 1431–1439 (2014). 24451622 10.1093/bioinformatics/btu029 PMC4016702

[30] Wu H, Xu T, Feng H Detection of differentially methylated regions from whole-genome bisulfite sequencing data without replicates. Nucleic Acids Res. 43(21), e141 (2015). 26184873 10.1093/nar/gkv715 PMC4666378

[31] Mclean CY, Bristor D, Hiller M GREAT improves functional interpretation of *cis*-regulatory regions. Nat. Biotechnol. 28(5), 495–501 (2010). 20436461 10.1038/nbt.1630 PMC4840234

[32] Xu Z, Taylor JA. Reliability of DNA methylation measures using Illumina methylation BeadChip. Epigenetics 16(5), 495–502 (2021). 32749174 10.1080/15592294.2020.1805692 PMC8078668

[33] Shvetsov YB, Song MA, Cai Q Intraindividual variation and short-term temporal trend in DNA methylation of human blood. Cancer Epidemiol. Biomarkers Prev. 24(3), 490–497 (2015). 25538225 10.1158/1055-9965.EPI-14-0853 PMC4355238

[34] Wang T, Guan W, Lin J A systematic study of normalization methods for Infinium 450K methylation data using whole-genome bisulfite sequencing data. Epigenetics 10(7), 662–669 (2015). 26036609 10.1080/15592294.2015.1057384 PMC4623491

[35] Pidsley R, Zotenko E, Peters TJ Critical evaluation of the Illumina MethylationEPIC BeadChip microarray for whole-genome DNA methylation profiling. Genome Biol. 17(1), 208 (2016). 27717381 10.1186/s13059-016-1066-1 PMC5055731

[36] Heiss JA, Brennan KJ, Baccarelli AA Battle of epigenetic proportions: comparing Illumina's EPIC methylation microarrays and TruSeq targeted bisulfite sequencing. Epigenetics 15(1-2), 174–182 (2020). 31538540 10.1080/15592294.2019.1656159 PMC6961683

[37] Shu C, Zhang X, Aouizerat BE, Xu K. Comparison of methylation capture sequencing and Infinium MethylationEPIC array in peripheral blood mononuclear cells. Epigenetics Chromatin 13(1), 51 (2020). 33228774 10.1186/s13072-020-00372-6 PMC7684759

[38] Yousefi P, Huen K, Dave V, Barcellos L, Eskenazi B, Holland N. Sex differences in DNA methylation assessed by 450 K BeadChip in newborns. BMC Genomics 16, 911 (2015). 26553366 10.1186/s12864-015-2034-y PMC4640166

[39] Martin E, Smeester L, Bommarito PA Sexual epigenetic dimorphism in the human placenta: implications for susceptibility during the prenatal period. Epigenomics 9(3), 267–278 (2017). 28234023 10.2217/epi-2016-0132 PMC5331919

[40] Solomon O, Huen K, Yousefi P Meta-analysis of epigenome-wide association studies in newborns and children show widespread sex differences in blood DNA methylation. Mutat. Res. Rev. Mutat. Res. 789, 108415 (2022). 35690418 10.1016/j.mrrev.2022.108415 PMC9623595

[41] Grant OA, Wang Y, Kumari M, Zabet NR, Schalkwyk L. Characterising sex differences of autosomal DNA methylation in whole blood using the Illumina EPIC array. Clin. Epigenetics 14(1), 62 (2022). 35568878 10.1186/s13148-022-01279-7 PMC9107695

[42] Solomon O, Macisaac J, Quach H Comparison of DNA methylation measured by Illumina 450K and EPIC BeadChips in blood of newborns and 14-year-old children. Epigenetics 13(6), 655–664 (2018). 30044683 10.1080/15592294.2018.1497386 PMC6140901

[43] Chen YA, Lemire M, Choufani S Discovery of cross-reactive probes and polymorphic CpGs in the Illumina Infinium HumanMethylation450 microarray. Epigenetics 8(2), 203–209 (2013). 23314698 10.4161/epi.23470 PMC3592906

[44] Labarre BA, Goncearenco A, Petrykowska HM MethylToSNP: identifying SNPs in Illumina DNA methylation array data. Epigenetics Chromatin 12(1), 79 (2019). 31861999 10.1186/s13072-019-0321-6 PMC6923858

[45] Yousefi P, Huen K, Aguilar Schall R Considerations for normalization of DNA methylation data by Illumina 450K BeadChip assay in population studies. Epigenetics 8(11), 1141–1152 (2013). 23959097 10.4161/epi.26037 PMC6242262

[46] Villicana S, Bell JT. Genetic impacts on DNA methylation: research findings and future perspectives. Genome Biol. 22(1), 127 (2021). 33931130 10.1186/s13059-021-02347-6 PMC8086086

[47] Mansell G, Gorrie-Stone TJ, Bao Y Guidance for DNA methylation studies: statistical insights from the Illumina EPIC array. BMC Genomics 20(1), 366 (2019). 31088362 10.1186/s12864-019-5761-7 PMC6518823

[48] Crabbe JC, Wahlsten D, Dudek BC. Genetics of mouse behavior: interactions with laboratory environment. Science 284(5420), 1670–1672 (1999). 10356397 10.1126/science.284.5420.1670

[49] Kobayashi Y, Inaba H, Iwakura Y Inter-breeder differences in prepulse inhibition deficits of C57BL/6J mice in a maternal immune activation model. Neuropsychopharmacol. Rep. 41(3), 416–421 (2021). 34043885 10.1002/npr2.12178 PMC8411318

[50] Von Kortzfleisch VT, Karp NA, Palme R, Kaiser S, Sachser N, Richter SH. Improving reproducibility in animal research by splitting the study population into several ‘mini-experiments’. Sci. Rep. 10(1), 16579 (2020). 33024165 10.1038/s41598-020-73503-4 PMC7538440

[51] Saffari A, Silver MJ, Zavattari P Estimation of a significance threshold for epigenome-wide association studies. Genet. Epidemiol. 42(1), 20–33 (2018). 29034560 10.1002/gepi.22086 PMC5813244

[52] Wessely F, Emes RD. Identification of DNA methylation biomarkers from Infinium arrays. Front. Genet. 3, 161 (2012). 22936948 10.3389/fgene.2012.00161 PMC3427494

