# Supplementary figures and images for: Beadchip technology to detect DNA methylation in mouse faithfully recapitulates whole-genome bisulfite sequencing
Source: Epigenomics. 2023 Apr 5;15(3):115–29. doi: 10.2217/epi-2023-0034 (PMC10131490; doi:10.2217/epi-2023-0034)

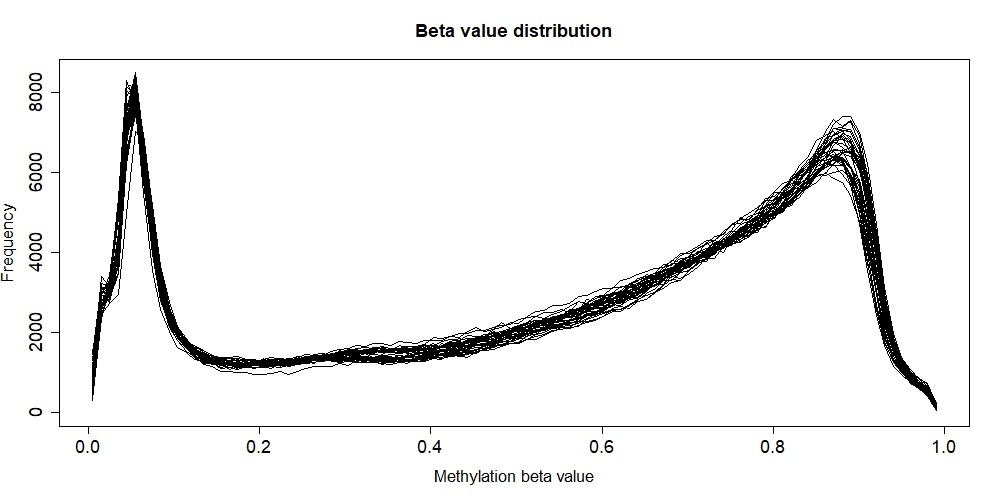

Supplement: Supplementary file 1 [file epi-15-115-s1.jpeg]

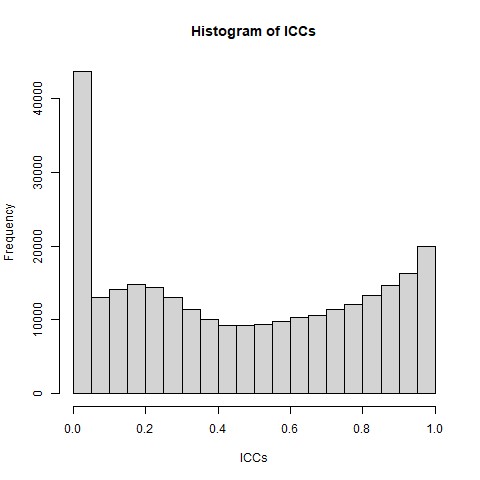

Supplement: Supplementary file 2 [file epi-15-115-s2.jpeg]

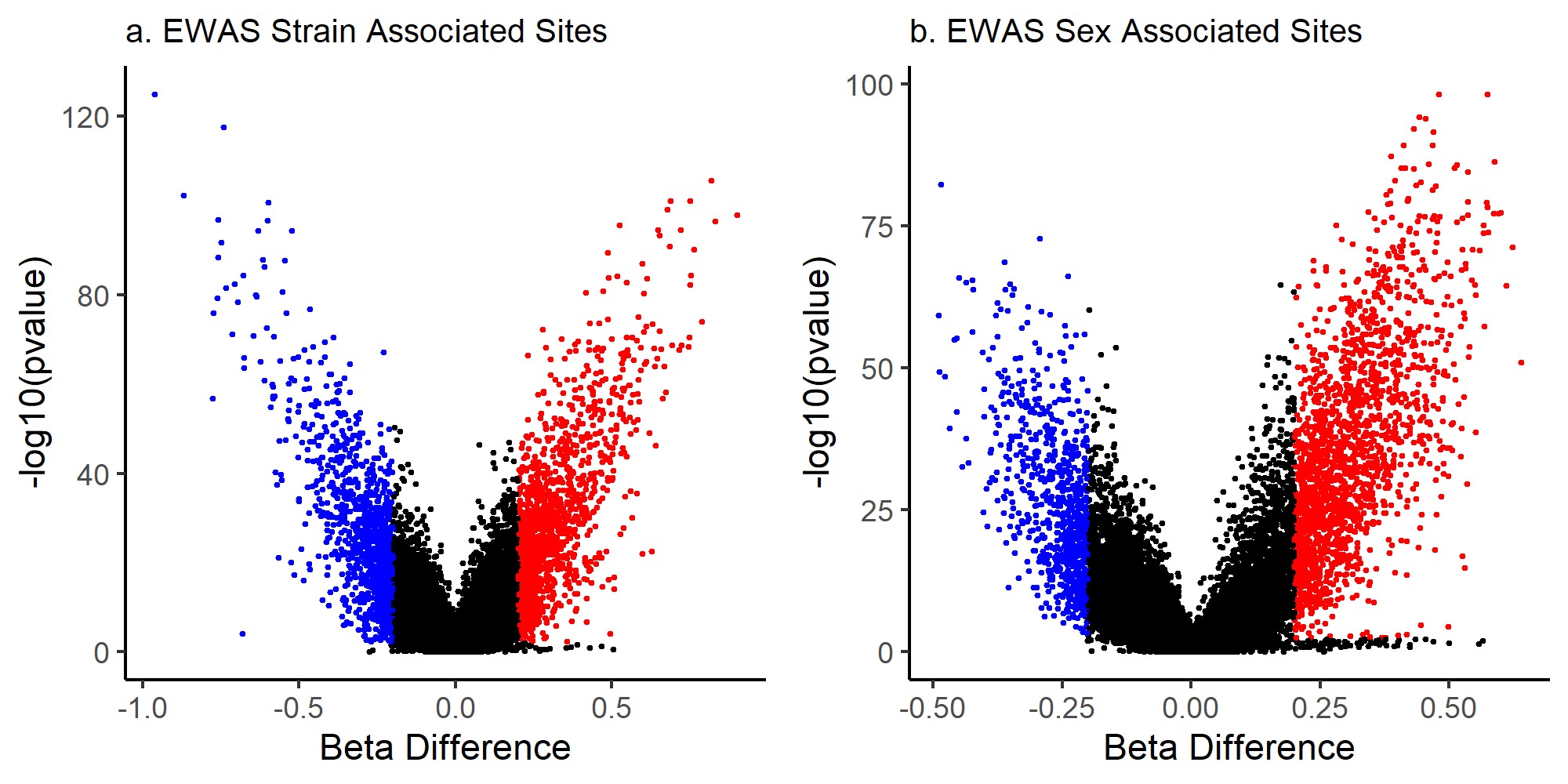

Supplement: Supplementary file 3 [file epi-15-115-s3.jpeg]
